# Ablative Radiotherapy (ART) for Locally Advanced Pancreatic Cancer (LAPC): Toward a New Paradigm?

**DOI:** 10.3390/life12040465

**Published:** 2022-03-22

**Authors:** Nicola Simoni, Gabriella Rossi, Francesco Cellini, Viviana Vitolo, Ester Orlandi, Vincenzo Valentini, Renzo Mazzarotto, Nicola Sverzellati, Nunziata D’Abbiero

**Affiliations:** 1 Radiotherapy Unit, Azienda Ospedaliera Universitaria, 43126 Parma, Italy; ndabbiero@ao.pr.it; 2 Department of Radiation Oncology, Azienda Ospedaliero Universitaria Integrata, 37126 Verona, Italy; gabriella.rossi@aovr.veneto.it (G.R.); renzo.mazzarotto@aovr.veneto.it (R.M.); 3 Radioterapia Oncologica ed Ematologia, Dipartimento Universitario Diagnostica per Immagini, Università Cattolica del Sacro Cuore, 00168 Roma, Italy; f.cellinimd@gmail.com (F.C.); vincenzo.valentini@policlinicogemelli.it (V.V.); 4 Radioterapia Oncologica ed Ematologia, Dipartimento di Diagnostica per Immagini, Fondazione Policlinico Universitario “A. Gemelli” IRCCS, 00168 Roma, Italy; 5 Radiation Oncology Clinical Department, National Center for Oncological Hadrontherapy (CNAO), 27100 Pavia, Italy; viviana.vitolo@cnao.it (V.V.); ester.orlandi@cnao.it (E.O.); 6 Division of Radiology, Azienda Ospedaliera Universitaria, 43126 Parma, Italy; nicola.sverzellati@unipr.it

**Keywords:** locally advanced, pancreatic cancer, ablative radiotherapy, intensive chemotherapy

## Abstract

Locally advanced pancreatic cancer (LAPC) represents a major urgency in oncology. Due to the massive involvement of the peripancreatic vessels, a curative-intent surgery is generally precluded. Historically, LAPC has been an indication for palliative systemic therapy. In recent years, with the introduction of intensive multi-agent chemotherapy regimens and aggressive surgical approaches, the survival of LAPC patients has significantly improved. In this complex and rapidly evolving scenario, the role of radiotherapy is still debated. The use of standard-dose conventional fractionated radiotherapy in LAPC has led to unsatisfactory oncological outcomes. However, technological advances in radiation therapy over recent years have definitively changed this paradigm. The use of ablative doses of radiotherapy, in association with image-guidance, respiratory organ-motion management, and adaptive protocols, has led to unprecedented results in terms of local control and survival. In this overview, principles, clinical applications, and current pitfalls of ablative radiotherapy (ART) as an emerging treatment option for LAPC are discussed.

## 1. Introduction

Pancreatic ductal adenocarcinoma (PDAC) is an aggressive disease, with a 5-year survival rate of less than 10% and increasing incidence [1,2]. At the time of diagnosis, 30–40% of patients present with unresectable locally advanced disease (LAPC), due to extensive involvement of adjacent major blood vessels that jeopardize curative-intent surgery [3]. This is mostly due to the elusive symptomatology at presentation and the lack of effective screening tests. According to current National Comprehensive Cancer Network (NCCN) guidelines, the definition of locally advanced disease includes lesions with: (1) encasement of the celiac axis (CA) or superior mesenteric artery (SMA) for head/uncinate process; (2) encasement of CA or SMA or contact with the CA and aortic involvement for body/tail, and (3) unreconstructible superior mesenteric vein (SMV)/portal vein (PV) due to tumor involvement or occlusion [4].

At present, the mainstay of treatment for LAPC is systemic chemotherapy with intensive multiagent regimens, such as leucovorin, fluorouracil, irinotecan, and oxaliplatin (FOLFIRINOX) or gemcitabine plus nab-paclitaxel (GNP), that have been demonstrated to improve survival in the metastatic and adjuvant subset [5,6,7]. Although the main cause of death is represented by the distant spread of disease, achieving a durable local control (LC) is of paramount importance, given that about one in three patients dies from complications related to uncontrolled local growth [8]. Thus, radiation therapy may represent a fundamental option to optimize LC in LAPC, potentially improving patients’ quality of life (QoL) and survival.

It is well-known that the radiation dose delivered to the target volume is crucial for tumor control. From a radiobiology standpoint, the concept of ablative radiotherapy (ART) generally involves the administration of a biologically effective dose (BED) of approximately 100 Gy (α/β tumor = 10), which is likely needed to achieve the complete eradication of viable tumor cells. Indeed, the relationship between higher BED_10_ and long-term LC (≥90%) has been widely demonstrated for lung and liver tumors [9,10,11]. However, to limit the risk of severe gastrointestinal toxicities, lower radiation dosing has generally been recommended in LAPC [12].

In recent years, impressive technological advances in radiotherapy have dramatically changed the approach to unresectable pancreatic cancer. A new paradigm is emerging, with a gradual transition from conventional standard-dose radiotherapy to advanced ablative-dose radiotherapy. The application of sculpted dose distributions with intensity-modulated radiotherapy (IMRT), stereotactic techniques, advanced tumor motion management, and sophisticated image guidance (IGRT), has allowed for the implementation of ART into clinical practice. These cutting-edge technologies enable extremely accurate delivery of radiation, thus allowing for the administration of more effective doses to the tumor, simultaneously minimizing the exposition of surrounding critical structures. In summary, the use of high precision delivery procedures represents a meaningful approach to improve cure rates by expanding the therapeutic index of radiotherapy [13].

This overview will focus on the growing evidence of the role of ablative radiotherapy (ART) in LAPC, as a promising alternative to the currently common practice of using standard-dose radiotherapy. Given the many unanswered questions, we will attempt to address the rationale, treatment strategies, clinical results, and challenges of ART for unresectable pancreatic cancer.

## 2. The Unresolved Dilemma of Standard-Dose Radiation Therapy

The role of radiation therapy in LAPC remains a matter of debate. Currently, the American Society for Radiation Oncology (ASTRO) clinical practice guidelines recommend for LAPC the administration of 50.4–56 Gy in 1.8–2.2 Gy/fraction with conventionally fractionated radiotherapy (CFRT), or alternatively, 33–40 Gy in five fractions if stereotactic body radiation therapy (SBRT) is adopted [14]. Overall, the biologically effective dose (BED_10_) thus administered corresponds to a range of approximately 60 to 70 Gy. However, the effectiveness of such standard dosing has been questioned.

Patients with LAPC have historically been treated with a combination of chemotherapy and conventionally fractionated radiotherapy. Randomized trials (RCTs) have compared standard-dose CFRT with concomitant chemotherapy versus chemotherapy alone with conflicting results [2,3,4,5,6]. The Gastrointestinal Tumor Study Group compared the survival of patients treated with streptozocin, mitomycin, and 5-fluorouracil (SMF) versus radiation combined with 5-fluorouracil followed by SMF [15]. The 1-year OS was 41% in the radio–chemotherapy group versus 19% in the chemotherapy alone group (*p* < 0.02). Later, in the Eastern Cooperative Oncology Group trial (ECOG 4201) a slight improvement in median OS (11.1 vs. 9.2 months; *p* = 0.017) was found with the use of concurrent radiation (50.4 Gy in 28 fractions) over gemcitabine alone [16]. On the other hand, the 1985 Eastern Cooperative Oncology Group study and the 2008 Fédération Francophone de Cancérologie Digestive and Société Française de Radiothérapie Oncologique (FFCD-SFRO) trials failed to demonstrate any improvement in survival with CFRT [17,18]. Furthermore, the radio–chemotherapy arms generally exhibited increased toxicity. However, it should be noted that radiotherapy delivery in these trials was suboptimal, due to the use of historic techniques and large fields of radiation.

In the modern LAP-07 phase III trial, LAPC patients without disease progression after 4 months of induction chemotherapy (with gemcitabine +/− erlotinib) were randomized to continue chemotherapy or to receive radio–chemotherapy (54 Gy/30 fractions with concomitant capecitabine) [19]. The median OS was not significantly different between the two groups (16.5 versus 15.2 months, *p* = 0.83). However, radiotherapy was associated with a decrease in loco-regional progression (32% vs. 46%, *p* = 0.04) and with a longer period without any treatment (6.1 vs. 3.7 months, *p* = 0.02). In addition, the rate of grade ≥ 3 toxicity did not differ between the 2 groups, except for a slight worsening in nausea incidence (5.9%).

In recent years, stereotactic body radiation therapy (SBRT) has emerged as a promising treatment option for LAPC. The potential advantages of SBRT over CFRT are: (1) to deliver higher doses per fraction, potentially overcoming tumor cells radio resistance; (2) to shorten the overall treatment time; (3) to avoid interruptions or delays in the administration of systemic therapy, and (4) to reduce possible acute toxicities (e.g., nausea, diarrhea, lymphopenia). In 2015, a landmark phase 2 multi-institutional trial demonstrated the feasibility of SBRT in LAPC [20]. Forty-nine patients received gemcitabine for 3 weeks followed by SBRT to a total dose of 33 Gy in five fractions (6.6 Gy per fraction) delivered over 1 to 2 weeks. After SBRT, patients continued to receive chemotherapy until disease progression or toxicity. SBRT resulted in favorable acute and late grade ≥2 gastrointestinal toxicities (2% and 11%, respectively). Median OS, 1-year freedom from local progression, and conversion surgery rate were 13.9 months, 78%, and 8%, respectively. Other studies confirmed the feasibility of a fractionated stereotactic approach in patients with unresectable disease [21,22,23,24,25,26]. Taken together, in these studies the median OS and 1-year LC rate ranged from 15 to 19 months and from 76.3% to 87%, respectively, and the occurrence of serious adverse events was reported between 0% and 8%. 

A comparison between SBRT and CFRT for the definitive treatment of LAPC was recently performed in an international systematic review and meta-analysis [27]. For SBRT, the median dose delivered was 30 Gy in five fractions, while for CFRT the prevalent schedule was 50.4 Gy in 28 fractions concurrent with gemcitabine. The use of SBRT resulted in a modest improvement in 2-year OS (26.9% versus 13.7%, *p* = 0.004) without any increase in grade ≥ 3 late toxicities (9.0% versus 10.1%, *p* = 0.49). Basically, fractionated SBRT at the standard dose appears as a reasonable alternative to CFRT for LAPC, providing acceptable LC, a favorable toxicity profile, and convenient treatment duration, with similar oncological outcomes. Recent series have confirmed these findings [28,29]. 

Overall, some perplexities about the use of standard-dose radiotherapy in LAPC remain. However, the debatable impact on OS of both CFRT and SBRT, compared to chemotherapy alone, must be interpreted with caution. Indeed, most of the reported studies used combinations of chemotherapy and radiotherapy which were largely inadequate. In addition, the use of effective dosing has often been sacrificed to reduce the risk of toxicity. From a radiation oncologist’s perspective, there is a clear need to administer higher BED, regardless of the number of fractions adopted, to achieve durable local control and impact on survival. This need is even more of a priority, the more effective the systemic therapies become.

## 3. Clinical Rationale for Dose-Escalation in LAPC

Although the natural history of LAPC differs from that of metastatic disease, it is important to note that the majority of patients develop distant progression in the first few months from diagnosis [30,31]. Thus, a first period of systemic therapy is crucial to evaluate tumor biology and responsiveness. Today, the initial therapeutic approach for LAPC involves the use of systemic multi-agent therapies, such as FOLFIRINOX or GNP, which have demonstrated a significant survival benefit compared to single-drug schemes [5,6,32]. In the meta-analysis by Suker et al., the use of FOLFIRINOX in selected LAPC patients produced a median OS of 24.2 months, significantly higher than that reported with the use of gemcitabine alone (6–13 months) [33]. Noteworthy, in the study, the pooled proportion of patients who received radiotherapy and surgery was 63.5% and 25.9%, respectively, underscoring the role of LC in survival improvement. Similarly, in the international, open-label, multicenter, phase II LAPACT trial, GNP was associated with a median OS and time to treatment failure significantly higher compared to historical data [34]. In the absence of consensus on which regimen is preferable and on the optimal chemotherapy duration, for LAPC, it is common practice to administer 3–6 months of FOLFIRINOX or GNP before considering, in case of disease response or stability, the introduction of radiotherapy.

Metastatic disease progression represents the main cause of morbidity and mortality in LAPC. However, a seminal Johns Hopkins University (JHU) rapid autopsy series demonstrated that about one-third of patients die from complications related to locally destructive pancreatic cancer [7]. This finding was confirmed in a retrospective analysis that investigated the pattern of disease progression in 244 LAPC patients treated with first-line chemotherapy [35]. Notably, 41% of patients died without evidence of distant metastases. Additionally, the pattern of progression analysis of 69 LAPC patients treated in a phase II trial with cetuximab, gemcitabine, and oxaliplatin followed by cetuximab, capecitabine, and radiation therapy (50.4 Gy in 28 fractions) showed that isolated local tumor progression leading to death mostly occurred between 16 and 31 months, representing a significant cause of late disease-related mortality [36]. Taken together, these results demonstrate that local tumor progression may represent a significant cause of death in long-surviving patients, regardless of the presence of metastases. Thus, by using ART, and optimizing patient selection, it can be postulated that the improved LC afforded by non-standard dosing radiation therapy can lead to an OS benefit, expanding the currently small cohort of long-surviving LAPC patients [37]. Of note, the use of BED_10_ ≥ 70 Gy was associated with significant survival improvement in LAPC patients treated with consolidative radio–chemotherapy following induction chemotherapy (17.8 vs. 15.0 months for BED ≤ 70 Gy) [38]. Similarly, in a recent series of 149 LAPC patients treated with multi-fraction SBRT after intensive chemotherapy, the adoption of doses ≥ 40 Gy (BED_10_ ≥ 72 Gy) showed a superior OS (23 versus 14 months, *p* = 0.0007) and PFS (13 versus 10 months, *p* = 0.007) compared to doses < 40 Gy [39]. Furthermore, the combination of FOLFIRINOX and SBRT ≥ 40 Gy provided the best oncological outcomes (OS 24 months) among the study population.

In addition to a potential cause of death, the locally advanced disease is responsible for frequent hospitalization, the need for interventional procedures, and intensive drug treatments. About 70% of patients present with jaundice, more than half experience uncontrolled pain in the abdomen and back, and up to 20% have duodenal invasion resulting in obstruction and bleeding [40,41]. Radiotherapy represents an effective strategy to reduce or prevent local symptoms in advanced pancreatic cancer [42]. Indeed, in a retrospective series evaluating the effectiveness of SBRT in inoperable patients, abdominal pain symptom relief was reported in 73% of cases [43]. Therefore, in LAPC patients, effective LC may translate in a durable reduction in local tumor burden, thus ensuring an improvement in QoL.

## 4. Ablative Radiation Therapy (ART): A New Paradigm

Ablative radiotherapy (ART) consists of administering a biologically effective dose (BED_10_) of 100 Gy to the tumor. In pancreatic cancer, delivering such high doses is a challenge. The main limitation is represented by the tolerance of the surrounding luminal organs at risk (OARs), primarily the duodenum, stomach, and bowel. The radiosensitivity of gastrointestinal OARs, as well as the uncertainty created by organ motion and day-to-day difference in luminal organ shape, can expose the patient to severe adverse events (e.g., perforation, bleeding). The risk represents a real concern, especially when hypofractionation is adopted. As stated above, novel radiation approaches have definitively changed this paradigm. Different strategies have been investigated to administer higher effective doses to the pancreatic tumor, improving oncological outcomes while maintaining an acceptable toxicity profile (Table 1).

**Table 1:** Selected clinical studies on the application of dose-escalated radiotherapy in LAPC.

Study Year [Ref]	Study Type N Patients	RT Technique and Dose	OS *	PFS *	LC °	Toxicity	Relevant Findings
Krishnan 2016 [38]	Retrospective 200 PC	Dose-escalated with SIB - 50.4 Gy/28 fx - 63–70 Gy/28 fx - 67.5 Gy/15 fx	17.8 mo BED > 70 Gy (vs. 15.0 mo BED ≤ 70 Gy)	8.6 mo BED > 70 Gy (vs. 5.3 mo BED ≤ 70 Gy)	LRRFS 7.3 mo	4% G3	- OS calculated from chemoradiation start - 3-year OS 31% BED > 70 Gy (vs. 9% BED ≤ 70 Gy) - Low toxicity rate not related to high-BED
Toesca 2020 [39]	Retrospective 149 LAPC	SBRT - 20–45 Gy/5 fx	16 mo	-	1-year LC 86% 2-year LC 78%	7% G3; 0.6% G4 (cholangitis), 0.6% G5 (GI bleeding)	- The combination of SBRT doses > 40 Gy and FFX showed a superior OS and PFS (24 and 14 months) - 5% of patients underwent tumor resection
Reyngold 2021 [44]	Retrospective 119 LAPC	HART - 67.5 Gy/15 fx - 75 Gy/25 fx	26.8 mo	13.2 mo	2-year LC from HART 62%	8% G3 GI bleeding	- 2-year OS from HART 38%
Rossi 2021 [45]	Retrospective 64 LAPC	SBRT/HART - 30 Gy/5 fx with 50 Gy SIB - 50.4 Gy/28 fx with 78.4 Gy SIB	29.7 mo	8.7 mo	78.1%	1.6% G4 GI bleeding	- Surgery performed in 26.6% of pts (median OS not reached)
Liauw 2020 [46]	Phase I/II 15 LAPC	Dose escalation design - 30, 37.5, 45 Gy/3 fx	23 mo	7 mo	80%	No dose-limiting toxicity	- G > 3 GI bleeding associated with tumor volume, dose heterogeneity inside the PTV, and duodenal dose
Courtney 2021 [47]	Phase I 30 pts (19 LAPC)	Dose escalation design - 40, 45, 50 Gy/5 fx	17.1 mo	-	85.8%	6.7% late G4–5	- Among LAPC median OS 19.0 mo
Rudra 2019 [48]	Retrospective 44 LAPC	MRgRT - 30–35 Gy/5 fractions - 40–55 Gy/25–28 fx - 40–52 Gy/5 fx - 50–67.5 Gy/10–15 fx	2-year OS 67% BED > 70 Gy vs. 30% BED ≤ 70 Gy	-	77% BED > 70 Gy vs. 57% BED ≤ 70 Gy	6.8% G3 (all in standard dose)	- High-dose radiation and duration of induction chemotherapy significantly correlated with OS on univariate analysis, but not on multivariate analysis
Hassanzadeh 2020 [49]	Phase I 44 LAPC	MRgRT - 50 Gy/5 fx	15.7 mo	12.4 mo	1-year LC 84.3%	4.6% late G3	- Tumor abutted or invaded OARs in 79.5% and 11.1% of cases - Reoptimization performed for 93% of all fractions
Chuong 2021 [50]	Retrospective 35 LAPC	MRgRT - 35–50 Gy/5 fx	9.8 mo from RT	7.9 mo from RT	1-year LC 87.8%	2.9% G3 acute and late	- Five fractions delivered in consecutive days to a median total dose of 50 Gy, with 120–130% hotspot - ENI delivered to 57.1% of patients - Median treatment time 83 min
Murphy 2019 [51]	Phase II 48 LAPC	50.4–58.8 Gy/28 fx + 10–20 Gy IORT	31.4 mo	17.5 mo	-	No G ≥ 3 RT-related	- All pts received FFX + Losartan - IORT: 10 Gy for resected tumors, 15 Gy if the tumor was not resected - Surgery performed in 66% of pts, with 88% R0 resection rate (median OS 33 mo)

LAPC: locally advanced pancreatic cancer; N: number; RT: radiotherapy; OS: overall survival; PFS: progression-free survival; LC: local control; PC: pancreatic cancer; SIB: simultaneous integrated boost; Gy: gray; fx: fractions; mo: months; BED: biologically effective dose; LRRFS: loco-regional recurrence-free survival; GI: gastrointestinal; FFX: FOLFIRINOX; SBRT: stereotactic body radiation therapy; HART: hypo fractionated ablative radiation therapy; PTV: planning target volume; MRgRT: MR-guided radiation therapy; OARs: organs at risk; ENI: elective nodal irradiation; IORT: intraoperative radiation therapy. * Median from diagnosis, unless otherwise specified. ° Overall, unless otherwise specified.

### 4.1. Hypofractionated Ablative Radiation Therapy (HART)

The basic concept of hypofractionated ablative radiation therapy (HART) is the prescription of ablative doses to the tumor while maintaining the radiobiological principle of fractionation. This means the administration of high effective doses, delivered in 15–25 fractions (3.0–4.5 Gy/fraction), abandoning the ultra-hypofractionation paradigm of SBRT [52]. From a radiobiology point of view, increasing the number of fractions allows for the reduction in the equivalent dose to the OARs. By combining this principle with the concept of intentional dose inhomogeneity of intensity-modulated techniques (dose painting or simultaneous integrated boost), it is possible to produce a targeted dose escalation without increasing the overall treatment time. In addition, the better conformity and rapid dose fall-out of intensity-modulated techniques may significantly reduce the rate of acute and late severe toxicity compared with conventional radiotherapy, even in a dose-escalated scenario [53].

**Figure 1:**
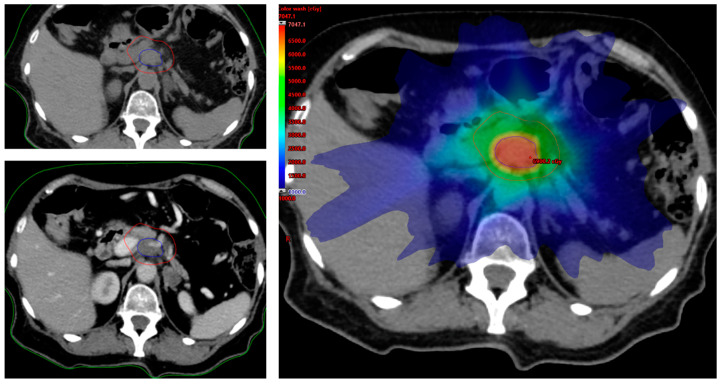
Hypofractionated Ablative Radiation Therapy (HART) contouring and plan. The high dose planning target volume (PTV_hd_, blue) encompasses the pancreatic lesion and tumor vessel interface inside the tumor planning target volume (PTV_t_, red). Dose prescription corresponds to 67.5 Gy and 37.5 Gy in 15 fractions to PTV_hd_ and PTV_t_, respectively.

The clinical efficacy of a fractionated dose-escalation approach in LAPC was first described in a retrospective series of the MD Anderson Cancer Center (MDACC) [38]. The analysis included 200 LAPC patients treated with induction chemotherapy followed by radio–chemotherapy. In relation to the distance from the gastrointestinal luminal organs (e.g., distance > 1 cm), 47 patients received escalated-dose radiation using a simultaneous integrated boost (SIB)-IMRT technique. A BED_10_ > 70 Gy was associated with improved survival (17.8 vs. 15.0 months, *p* = 0.03) and loco-regional recurrence-free survival (10.2 vs. 6.2 months, *p* = 0.05), as compared with BED_10_ ≤ 70 Gy. Remarkably, on multivariate analysis, BED_10_ resulted as the only independent predictor of survival (hazard ratio 0.63, *p* = 0.03), while the adverse events rate did not differ between the two groups. A larger series has confirmed these findings [54]. In addition, Passoni et al. demonstrated that the delivery of a dose of 44.25 Gy in 15 fractions to the whole tumor with a SIB up to 58 Gy to the tumor vessel interface (TVI), corresponding to a BED > 80 Gy, was feasible without reaching dose-limiting toxicity [55]. The dosimetric feasibility of HART in LAPC has been adequately demonstrated [56,57]. An example of a HART plan is shown in Figure 1.

In 2021, Reyngold et al. reported the impressive results of 119 patients with localized unresectable or medically inoperable pancreatic cancer treated with HART at the Memorial Sloan Kettering Cancer Center (MSKCC) regional network [44]. Induction systemic therapy was administered in 97.5% of patients, mostly consisting of FOLFIRINOX or GNP. HART consisted of 75 Gy/25 fractions and 67.5 Gy/15 fractions (BED_10_ = 98 Gy) in 81% and 19% of patients, respectively, both delivered with concurrent chemotherapy. Respiratory gating was used for respiratory motion management, and daily cone-beam computed tomography (CBCT) image guidance with selective adaptive planning was adopted to improve treatment accuracy. Median OS and PFS from diagnosis were 26.8 months and 13.2 months, respectively, significantly higher than those reported with standard-dose radiotherapy. The 2-year cumulative incidence of loco-regional progression was 32.8%; no grade ≥ 4 adverse events related to HART were reported. Notably, the results are consistent with those reported in a retrospective series of LAPC patients treated with 78.4 Gy in 28 fractions (BED_10_ 100 Gy) concomitant with chemotherapy at the Verona Hospital University [45].

In this context, a direct comparison between HART and surgery was recently performed in a retrospective analysis, including 209 pancreatic lesions with vascular involvement at the time of diagnosis [58]. All patients received induction chemotherapy, followed by HART in 49.8% or curative-intent resection in 50.2% of cases. The 18-months loco-regional control rate was similar between two groups (84% versus 79%, *p* = 0.252). In contrast, HART was associated with worse survival and distant progression rate. However, it is important to underline that in the HART group, patients presented with more extended tumors (70% of locally advanced disease, compared to 19.0% of surgery group, according to the AHPBA/SSO/SSAT resectability classification [59]), higher comorbidity rate, and poorer functional status, a selection bias that may partly justify these findings.

Undoubtedly, HART represents a promising strategy for safely delivering ablative doses in LAPC patients pretreated with intensive chemotherapy, providing loco-regional disease control similar to that associated with resection and encouraging survival. However, to date, data in support of HART derive mainly from retrospective experiences of single, high-volume centers, highly specialized in the treatment of pancreatic cancer. Furthermore, what the optimal radiotherapy schedule (15 versus 25 fractions), as well as the preferable chemotherapy combination, might be, are still open questions. Surely, HART represents a strategy to administer ablative doses even in those cases are not suitable for ultra-hypofractionation (e.g., wide contact or infiltration of luminal OARs) at the cost of longer treatment duration. Although a detailed discussion is beyond the scope of this review, the main technical considerations for prescribing ablative doses of radiotherapy with different fractionations are summarized in Table 2.

**Table 2:** Technical considerations for prescribing ablative doses of radiotherapy with different fractionations.

	HART		SABR
Dose/fractionation	75 Gy/25 fractions or 67.5 Gy/15 fractions BED 98 Gy Delivered on consecutive days		50 Gy/5 fractions BED 100 Gy Delivered on consecutive days or every other day Not recommended in case of direct invasion of GI tract
Target volume definition	Two dose levels (PTVhd and PTVt) with SIB [57] A SIB/SIP approach (3 dose level) can be adopted for SABR [45,60]		
PTV_hd_	GTV + TVI + 0–5 mm Subtracting overlap with PRV GI OARs		GTV + TVI + 0–3 mm Subtracting overlap with PRV GI OARs
PTVt	GTV + TVI + 5–10 mm Alternatively, PTV_t_ = CTV (GTV + TVI + CA/SMA + SMPV) + 5–10 mm		PTV_t_ = GTV + TVI + 3–5 mm
Nodal coverage	Proximal nodes permitted (e.g., CA, SMA, CHA, SMPV) ENI no longer recommended		Inclusion of perilesional nodal disease in selected patients
Dose prescription	PTV_hd_ 75 Gy/25 fractions or 67.5 Gy/15 fractions PTV_t_ 50 Gy/25 fractions or 37.5–42 Gy/15 fractions		PTV_hd_ 50 Gy/5 fractions PTV_t_ 33–40 Gy/5 fractions
Concomitant chemotherapy	Recommended (at radiation oncology discretion, capecitabine or gemcitabine)		Not recommended
Suggested OARs dose constraints	75 Gy/25 fractions Spinal cord D_max_ < 45 Gy PRV duodenum/stomach D_max_ < 60 Gy PRV bowel D_max_ < 54 Gy Kidneys D_mean_ < 18 Gy Liver D_mean_ < 30 Gy, V30 < 30 Gy	67.5 Gy/15 fractions Spinal cord D_max_ < 30 Gy PRV duodenum/bowel/stomach D_max_ < 45 Gy Kidneys V20 < 30% Liver D_mean_ < 24 Gy, 700 cc < 24 Gy	50 Gy/5 fractions Spinal cord D_max_ < 20 Gy PRV duodenum/bowel/stomach D_max_ < 35 Gy, V30 Gy < 5 cc, D_mean_ < 20 Gy Kidneys D_mean_ < 10 Gy, 200 cc < 17.5 Gy Liver 700 cc < 21 Gy
Planning	IMRT or VMAT with SIB		
Organ motion management	BH (DIBH or EEBH) 4D-CT Abdominal compression		
IGRT	Fiducial markers insertion Daily CBCT (Adaptive)		

HART: Hypo fractionated Ablative Radiation Therapy; SABR: Stereotactic Ablative Radiation Therapy; Gy: gray; BED: biologically effective dose; GI: gastrointestinal; PTVhd: high dose planning target volume; PTVt: tumor planning target volume; SIB: simultaneous integrated boost; SIP: simultaneous integrated protection; GTV: gross tumor volume; TVI: tumor-vessel interface; PRV OAR: planning organ at risk volume; CTV: clinical target volume; CA: celiac axis; SMA: superior mesenteric artery; SMPV: superior mesenteric-portal venous confluence; CHA: common hepatic artery; ENI: elective nodal irradiation; IMRT: intensity-modulated radiotherapy; VMAT: volumetric-modulated radiotherapy; BH: breath-hold; DIBH: deep inspiration breath-hold; EEBH: end-expiration breath-hold; 4D-CT: four-dimensional computed tomography; CBCT: cone-beam computed tomography.

### 4.2. Stereotactic Ablative Radiation Therapy (SABR)

Stereotactic Body Radiation Therapy (SBRT) is an external beam radiotherapy strategy in which a well-defined extracranial target is treated with a higher dose per fraction, compared to CFRT, delivered with precision and accuracy in five fractions or less [61]. When SBRT employs tumor-ablative doses, it is also referred to as Stereotactic Ablative Radiation Therapy (SABR) [62]. A 3-fraction SBRT regimen using ablative doses in a phase II trial was reported in 2005 but deemed unsafe due to severe toxicity within 2 weeks of treatment [63]. Similarly, early studies using radiosurgery (e.g., 25 Gy/single dose) reported unacceptable gastrointestinal adverse events [64]. However, in the last decade, the use of lower doses fractionated SBRT (e.g., 30–33 Gy in five fractions), in association with advances in radiation techniques, has been associated with acceptable oncological results, with a favorable toxicity profile [27]. More recently, SBRT has been advocated as a reliable strategy for potentially ablative purposes.

Because of the lack of standardization about SBRT dose and fractionation in pancreatic cancer, a recent study tried to compare the various regimens used in literature, converting all of them into 3-fraction equivalent doses [65]. For LAPC patients, the pooled model suggests 1-year local control of 79–86% in regimens equivalent to 30–36 Gy/3 fractions, showing, in the meanwhile, a decrease to less than 70% at doses below 24 Gy/3 fractions. That is why current efforts by radiation oncologists are addressed to dose-escalation studies, supported by advancements in imaging and radiation treatment planning. In this context, the dosimetric feasibility of ablative dosing SBRT in LAPC has been widely demonstrated [57,66,67].

In a recent retrospective series, 52 LAPC patients were treated with SBRT following intensive chemotherapy with FOLFIRINOX or GNP, administering 30 Gy in five fractions to the tumor volume and 50 Gy SIB (BED_10_ 100 Gy) to the vascular involvement [45]. Median OS and PFS in non-resected patients were 27.4 and 5.7 months, respectively, without relevant toxicity. Interestingly, surgery was performed on 26.6% of patients, with an R0 resection rate of 64.7%. In addition, in a prospective trial of SABR (30–45 Gy/3 fractions) for unresectable pancreatic cancer, the Authors found a median survival of 23 months from the time of diagnosis [46]. Notably, patients were enrolled regardless of the number of cycles and systemic therapy schedule, emphasizing the potential magnitude of benefit of using ablative doses. As further confirmation, in a retrospective analysis by Toesca et al. the combination of SBRT doses ≥ 40 Gy and FOLFIRINOX showed superior survival outcomes compared to the use of gemcitabine-based chemotherapy and SBRT doses < 40 Gy (1-year PFS and OS of 67% and 90% compared to 35% and 59%, respectively) [39].

However, in a recent phase I dose-escalation trial testing doses of 40, 45, and 50 Gy in five fractions, a nontrivial rate of severe late gastrointestinal toxicity, potentially attributable to radiation, was reported [47]. In particular, 6.7% of patients experienced G4–5 late toxicity, both of which occurred in the 45 Gy group. This finding reaffirms, if necessary, the delicate benefit–risk ratio when ultra-hypofractionation is adopted. Since SBRT is limited by gastrointestinal tract toxicity, current phase I-II trials are evaluating the addition of selective superoxide dismutase mimetic to expand the therapeutic window of SBRT [68,69]. Another strategy investigated to deliver ablative doses of radiation is the use of a sequential hypo fractionated radiotherapy boost (HRB) on primary pancreatic lesions after conventional CFRT. In a recent study, 31 patients affected by LAPC underwent gemcitabine- or capecitabine-based radio–chemotherapy (50.4 Gy in 28 fractions) preceded and/or followed by chemotherapy (gemcitabine alone or FOLFIRINOX schedule) [70]. An HRB on the GTV was administered within 1–13 months since the last therapy, with a prescription dose of 20–30 Gy in five fractions, based on the duodenal constraint. Interestingly, the 1-year LC and 2-year OS after HRB were 66.4% and 57.4%, respectively.

In conclusion, ablative dosing SBRT (or SABR) has demonstrated promising results in LAPC, yet at present, the optimal schedule must be determined. SBRT depends on accurate target definition, precise and reproducible patient set-up, and sophisticated organ motion management. Advances in radiation delivery, image-guidance (IGRT), and treatment planning may allow for dose escalation to levels not previously achievable, potentially improving LC and survival. On the other hand, the risk of severe gastrointestinal adverse events remains a concern, in particular, for lesions with a wide contact with luminal OARs. For patients with a tumor > 5–6 cm in its greatest dimension, significant nodal spread, or infiltrating duodenum or stomach, the adoption of HART may be preferable. In addition, the use of magnetic resonance-guided radiation therapy (MRgRT), allowing daily online adaptation and real-time organ motion management, represents a meaningful modern strategy to deliver ablative dosing SBRT.

### 4.3. MR-Guided Radiation Therapy (MRgRT)

A limit to widely deliver ART is represented by the risk of toxicity to the OARs surrounding the pancreatic lesion. The duodenum is usually the most dose-limiting organ, since a dose-dependent correlation with the frequency of grade ≥ 2 toxicity has been reported [71,72], even if the most advanced robotic radiosurgery techniques are used [73]. The management of HART and SBRT treatments is also conditioned by the difficulty of accurately identifying the therapy volumes, due to a limited soft-tissue definition of the images that are usually used in the abdomen, such as computed tomography (CT) and cone-beam computed tomography (CBCT) [74,75]. An innovative approach to the radiation oncological management of pancreatic cancer is represented by the integration of magnetic resonance (MR) imaging on-board by the so-called MR-hybrid machines. Linac accelerators are built integrated with MR scanners to increase some opportunities of the processes of simulation, planning, and delivery. The MR-guided RT (MRgRT) is currently provided by the main machines: Unity^®^ (Elekta, Stockholm, Sweden) uses a 1.5 Tesla MRI scanner with a 7 MV Flattening Filter Free (FFF) Linac; the MRIdian^®^ system (ViewRay, Cleveland, Ohio) applies a 0.35 Tesla MRI scanner with a 6 MV FFF Linac.

Currently, such systems deliver radiation therapy through the step-and-shoot application of intensity modulation (IMRT) and do not perform “sliding windows” IMRT or its evolution in volumetric modulated arc radiotherapy (VMAT). Since these applications are very useful for optimizing the dose distribution, that can theoretically represent a limit [76]. Nevertheless, the better on-board-image guidance provided by MR imaging rather than the CBCT and the chance to apply MR gating to treatment sessions has exclusive advantages for daily individuation of the target and OARs. The MRIdian^®^ system is currently the most widely adopted and clinically applied. As a consequence of that, gating by direct and fiducial-less visualization of the structures of interest (avoiding the use of surrogates to focus the gating system on) increases the versatility of this device to gating both the target and OARs. The gating can be personalized, modifying some of the treatment parameters at each fraction. Treatment gating protocols can be directly applied to target volumes, to surrogate target volumes (especially if the target is not clearly visible on positioning images) [77], or even to OARs in order to optimize their sparing [78].

Particularly when dealing with LAPC, the most relevant technical peculiarity of such systems is the ability to perform “online adaptive RT”. Targets and OARs are re-contoured before the RT session while the patient lays on the treatment couch, obtaining a prediction of the dose distribution adapted to the daily changes of anatomy and, if needed, an optimized plan can be reloaded and then delivered. All these potential advantages can be exploited to prescribe a higher biological dose while avoiding undue high doses to adjacent critical organs, such as the duodenum, stomach, and bowel [79,80]. The feasibility of MRgRT for pancreatic cancer has been evaluated in a retrospective series oriented toward technical applications of the approach, with good performing results in terms of safety and feasibility [81,82].

A retrospective multicentric analysis on 44 patients with pancreatic cancer treated with MRgRT was published by Rudra et al. [48]. They included patients with different presentations: borderline resectable, locally advanced, and medically inoperable pancreatic cancer. Patients were treated with various approaches: conventional fractionation (40–55 Gy in 25–28 fractions), hypofractionation (50–67.5 Gy in 10–15 fractions), and SBRT (30–35 Gy in five fractions; 40–52 Gy in five fractions). Notably, adaptive MRgRT treatments were delivered to patients receiving 15 or fewer fractions. Patients were stratified into two groups depending on the BED_10_: high-dose (BED_10_ > 70 Gy) and lower-dose groups (BED_10_ ≤ 70 Gy). With a median follow-up of 17 months, BED_10_ > 70 Gy patients (24; 55%) showed significantly improved 2-year OS (49 vs. 30%, *p* = 0.03) compared with the BED_10_ ≤ 70 Gy patients, confirming the clinical impact of an adequate dose. Moreover, gastrointestinal severe toxicity (e.g., grade over 3) occurred only in three patients in the standard-dose group and did not occur in the high-dose group. These results suggest the potential of MRgRT to help deliver a dose more conformal to the initially planned (since 31 out of 44 patients had been on-line adapted) providing quite tolerable radiation treatments and confirming the impact of adequate BED on treatment outcome for pancreatic cancer. Thus, the role of MRgRT could be particularly promising for LAPC presentations, at which the highest possible BED should be aimed, given the improbability of the chance for the conversion to a resectable presentation. A retrospective series evaluated the benefit of using stereotactic magnetic resonance-guided adaptive radiation therapy (SMART) with 50 Gy in five fractions (BED_10_ 100 Gy) in 44 LAPCs [49]. Reoptimization was performed for 93% of all fractions. One-year local control was 84.3%, while OS was 15.7 months, with a very acceptable toxicity rate. More recently, Chuong et al. demonstrated excellent LC and toxicity rates with SMART at ablative dosing [50]. Interestingly, elective nodal irradiation to include a 5–10 mm radial expansion around the celiac axis, superior mesenteric vein, and the superior mesenteric artery was adopted in 57.1% of patients (Table 1).

In summary, MRgRT can definitively allow for safer dose delivery, particularly for SBRT in pancreatic cancer, and can allow for safer dose escalation. Whether MRgRT in itself can provide superior results over those of a standard Linac for PDAC has still not been evaluated or reported in the literature, to the best of our knowledge. A multi-institutional prospective trial is ongoing among US Centers [83]. The inclusion of 133 patients is expected, delivering 50 Gy in five fractions (BED_10_ = 100 Gy) to the target lesion. The primary endpoint is grade 3 or higher acute toxicity; secondary endpoints include OS, distant progression-free survival, and quality of life (QoL).

### 4.4. Intraoperative Radiation Therapy (IORT)

Intraoperative radiation therapy (IORT) is defined as the application of a single high dose of irradiation at the time of surgery. In pancreatic adenocarcinoma, IORT is typically used as a boost to the tumor bed after external beam radiotherapy (EBRT) and resection, or to the tumor in situ in case of unresectable disease [84]. The potential advantages of IORT include: (1) the surgical exclusion of radiosensitive OARs that are displaced away from the radiation field; (2) the direct visualization of the target lesion with targeted delivery of high-dose radiation, and (3) the reduction in intra-fraction uncertainties due to organ motion. Thereby, IORT can allow for a dose-escalation purpose, increasing the cumulative BED administered to the tumor, potentially improving the therapeutic ratio compared with EBRT alone without worsening the toxicity profile [85]. On the other hand, the possible drawbacks of IORT are represented by the limited diffusion of equipment and dedicated surgical suite, the need for close collaboration between radiation oncologists and surgeons, as well as the lack of three-dimensional treatment planning.

Historical studies of IORT in LAPC reported excellent symptoms control, in the absence of relevant radiation-related adverse events [86,87]. A study from the Mayo Clinic compared the survival outcomes of LAPC patients treated with EBRT alone (N = 122, 40–60 Gy CFRT) or EBRT + IORT (N = 37, 45–55 Gy CFRT + 20 Gy IORT) [88]. The addition of IORT resulted in a 2-year LC of 66% compared to 20% with EBRT alone (*p* < 0.001), underscoring the importance of the cumulative effective dose administered to the tumor. In the study, the increased LC did not translate into better survival; however, it is important to note that patients only received 5-fluorouracil as single-drug systemic therapy. With the advent of more effective intensive chemotherapy schemes, and sophisticated delivery methods for external beam radiotherapy (e.g., image-guided intensity-modulated and stereotactic radiotherapy), IORT has been rediscovered [51].

In 2020, the Massachusetts General Hospital (MGH) group reported the results of a retrospective analysis of 132 patients with borderline resectable or locally advanced disease (BR/LAPC) treated with FOLFIRINOX and chemo–radiotherapy, followed by surgical exploration in an IORT-equipped operating room [89]. Specifically, following 4 months of chemotherapy, patients received consolidative EBRT at 50.4–58.8 Gy in 28 fractions. Finally, 65% of patients underwent surgery with IORT, while 35% were found with unresectable lesions at surgical exploration and received IORT alone. The median OS and PFS for resected + IORT versus IORT alone patients were 46.7 and 21.5 versus 23 and 14.7 months, respectively (*p* < 0.01). Remarkably, in the IORT alone group, the 2-year OS rate was 49.1% and local failure occurred in only 15% of patients. Significantly, the two-year survival rate reaching 50% in non-resected patients is consistent with the results of HART and SABR [44,45].

Overall, IORT seems a risk-adapted, accurate, and efficient radiation dose-escalation strategy to safely administer a precise ultra-boost of irradiation directly to the unresected tumor removing the surrounding critical OARs or to sterilize a tumor bed at high risk of microscopically positive margins (R1) and local recurrence. Current ESTRO IORT Task Force/ACROP recommendations suggest the application of doses in the range of 10.0 to 12.5 Gy for completely resected tumors, 12.5–15 Gy if the residual microscopic malignant disease is suspected, and 15–20 Gy for unresected lesions [90,91]. For LAPC, considering the sum of the standard-dose EBRT + IORT boost, a total BED > 100 Gy is thus administered to the tumor. The phase II PACER trial is currently exploring the role of IORT in treating unresectable pancreatic cancer after 3–6 months of chemotherapy and external radiation therapy, including both CFRT and SBRT [92].

### 4.5. Particle Therapy

A new frontier in the era of advanced radiotherapy techniques is represented by particle therapy where charged nuclear particles, both protons and carbon ions, thanks to their physical intrinsic selectivity, are able to deposit most of their energy at a specific depth, known as the Bragg peak, with no exit dose behind the target [93]. This allows escalating the dose to the tumor, improving the therapeutic ratio, and delivering a lower dose to surrounding healthy tissues, with lower related NTCP. The relative biological effectiveness (RBE) of proton therapy (PT) is reported to be only 10% higher than X-rays. Conversely, carbon ions (CIRT), thanks to a mass larger than protons, which are able to generate dense ionizations in their track, allowing breaks in cellular DNA with a superior tumoricidal effect and biological effectiveness two to four times higher compared with X-rays [94,95].

Clinical experience with proton therapy in pancreatic cancer is still limited to a mono-institutional series from Japan and the United States with limited numbers of treated patients. Dose and fractionation employed regimens range from 50 Gy RBE to 67.5 Gy RBE in 25 fractions, with higher dose per fractions in the case of a favorable anatomical positioning between tumor location and bowel loops, with a target volume including elective nodal irradiation, and with chemotherapy concomitant to radiation therapy. With this approach, Hiroshima et al. reported a retrospective series of 42 LAPC patients treated with concurrent chemo (gemcitabine or S1)—proton therapy [96]. With a median follow up of 14 months, 1- and 2-year OS rates were 77.8 and 50.8%, respectively, with a median survival time of 25.6 months. Notably, LC rate at 1 and 2 years was reported, respectively, as 90.1 and 76.7%, with a median time to local recurrence of more than 36 months. No grade 3 or higher late adverse effects were described. By contrast, less favorable experience in terms of gastrointestinal toxicity was described in a phase I/II study by Terashima and colleagues in 50 LAPC patients treated with proton radiotherapy and concomitant Gemcitabine [97]. In the group of patients treated with doses ranging from 67.5 to 70.2 Gy (RBE) a toxicity rate G > 3, including gastric ulcer and hemorrhage, were reported in five cases (10%). 

CIRT experience comes from Japan where this innovative field of research was first developed. Shinoto and colleagues, with the intent to define the maximum tolerated dose of CIRT in association with chemotherapy, performed a dose-escalation trial in 72 LAPC patients treated with up to 55.2 Gy [RBE], in 12 fractions with concurrent Gemcitabine (1000 mg/m^2^) [98]. The Authors found better outcomes in the subgroup of patients receiving at least 45.6 Gy [RBE], with a 2-year local recurrence rate reported at 17% and a 2-year survival rate of 54%. Further, 1- and 2-year OS rates in all patients were, respectively, 73% and 35%, and the median OS was 19.6 months. Despite the association with chemotherapy, no significant gastrointestinal toxicity was described and a greater than G3 (ulcer) was seen in only one patient (1%). Such results in terms of outcome and toxicity were independently confirmed by three Japanese centers in the retrospective multi-institutional study of the “Japan Carbon ion Radiation Oncology Study Group (J-CROS) [99]. The three institutions, using analogous treatment approaches in terms of selection criteria, prescription doses (52.8 Gy [RBE] or 55.2 Gy [RBE], in 12 fractions), and target volumes (including ENI), achieved a median OS of 21.5 months, with 1- and 2-year OS rate being, respectively, 73% and 46%. Among the 72 patients enrolled, only one patient (1%) developed a grade 3 duodenal ulcer, while no grade 4–5 toxicity was reported. Recently, data from the same study were updated. One hundred and forty-four patients treated with 55.2 Gy RBE from 2014–2018 confirmed data on the 1-, 2-, and 4-year survival rate of 47%, 25%, and 18%, respectively [100]. Chemotherapy with Gemcitabine or S-1 was associated with radiation therapy in 86% of patients; 9% G3 gastro-intestinal toxicity (ulceration) was reported.

A phase I, dose-escalation study from the Albert Einstein College of Medicine and the Shanghai Proton and Heavy Ion Center has recently been completed, with the accrual of 14 LAPC patients [101]. The study used a mixed beam approach with photon and CIRT in two parallel cohorts of patients (with tumor distance within or major than 5 mm), with the carbon dose progressively increasing until it was 56 GyRBE in 4 Gy RBE/fractions, starting from 3 Gy RBE/fraction. Results are awaited. Furthermore, the Shanghai Proton and Heavy Ion Center is evaluating in LAPC the safety of the delivery of a total dose of 51 Gy RBE on the clinical target volume, with a simultaneous integrated boost of 59.5 to 62.9 Gy RBE to the GTV in the same 17 fractions [102]. Another prospective, single-center phase I/II trial was registered by the Heidelberg Ion Therapy Center (HIT) [103]. 

In conclusion, to date, there is no definitive evidence of the superiority of particle therapy for LAPC compared to other ART modalities because of few data, and incomparable experiences from different institutions. Nevertheless, particle therapy represents an advantageous treatment opportunity to better investigate, possible in well-designed phase III trials.

## 5. Integrating ART in Total Neoadjuvant Therapy (TNT)

Surgery has historically been considered the only chance of cure for pancreatic–adeno carcinoma. Thus, a scenario including neoadjuvant therapy followed by conversion surgery has increasingly been tested in primarily unresectable disease [104]. In particular, the introduction of multi-agent chemotherapy has facilitated potential resection with curative intent in selected LAPC patients with excellent outcomes [105,106]. In recent years, total neoadjuvant treatment (TNT), including preoperative chemotherapy and (chemo-)radiotherapy, has emerged as a promising strategy for locally advanced tumors to optimize the delivery of a trimodality approach [107,108]. Potential advantages of TNT include better tolerability and adherence to treatment, the administration of full doses of chemotherapy to treat occult metastases early, and tumor downsizing and downstaging to increase the likelihood of radical resection, making it theoretically very positive for LAPC. However, although in the FOLFIRINOX era, the addition of preoperative radiotherapy has been shown to potentially increase the likelihood of radical resection, pathological response rates, loco-regional control, and survival compared to chemotherapy alone [109], the optimal neoadjuvant strategy in LAPC has yet to be determined [110].

A further critical topic of debate is whether and how ablative-dose radiotherapy (ART) can be declined in a TNT scenario in LAPC. Two different strategies should be considered. First, ART by using HART or SABR may represent a meaningful therapeutic option for maximizing oncological outcomes, by achieving the complete eradication of viable tumor cells survived after chemotherapy. A concern related to using ART as a neoadjuvant approach could be related to the possible worsening of surgical complications caused by using non-standard doses for a preoperative setting. However, a recent series has demonstrated the feasibility of conversion curative-intent surgery after ART. In a retrospective analysis by the Verona Hospital University, major surgical complications after SABR were reported in 17.1% of cases, with a rate of delayed gastric emptying, clinically relevant pancreatic fistula, and post-pancreatectomy hemorrhage of 8.5%, 5.7%, and 5.7%, respectively, that are consistent to those reported with preoperative CFRT [45]. Remarkably, median OS in resected patients has not been reached. The phase II MAIBE trial is underway to further explore the role of HART, in combination with chemotherapy, to improve the chance of surgery and long-term survival [111].

A second strategy involves the use of an IORT boost. In a single-arm phase 2 clinical trial conducted at MGH, 49 LAPC patients underwent TNT, including FOLFIRINOX e losartan, chemoradiation to 50.4 to 58.8 Gy, and IORT with and without resection [87]. Notably, for the 34 patients who underwent resection, median OS and PFS were 33.0 and 21.3 months, respectively, without limiting toxicities. More recently, the impact of adding IORT to surgery in BR/LAPC patients after FOLFIRINOX and chemoradiation was retrospectively analyzed [112]. Among no-IORT patients, R1 resection negatively impacted survival; in contrast, in the IORT group, no difference between R0 vs. R1 resection status was found for OS (R0 48 months vs. R1 37 months; *p* = 0.307) and PFS (R0 29 months vs. R1 20 months; *p* = 0.114). The study demonstrated that the use of IORT could mitigate the prognostic negative effect of an R1 resection on survival outcomes of pancreatic cancer patients, without increasing postoperative complications.

As such, limited data on TNT in LAPC show promising clinical results, but further studies should be performed to clarify the optimal integration and timing of chemotherapy and radiotherapy. The use of ablative doses in the neoadjuvant setting does not seem to worsen the surgical outcomes. However, although representing an intriguing strategy to optimize survival, at present the association of ART and surgery should be performed in clinical trials or at experienced, high-volume centers.

## 6. The Borderline Resectable Disease

The current definition of Borderline Resectable Pancreatic Cancer (BRPC) involves three different parameters: anatomical, biological, and conditional [113]. Anatomic factors include vascular “abutment”, which generally indicates <180° of solid tumor contact around the peripancreatic vessels (CA, SMA, CHA, SMV/PV), or vein involvement allowing for safe and complete resection and reconstruction [4]. Biological factors include the presence of regional lymph nodes metastases or a serum carbohydrate antigen (CA) 19–9 level > 500 units/mL, while a conditional factor is represented by a depressed Eastern Cooperative Oncology Group (ECOG) performance status (2 or more). Tumors classified as BRPC, although potentially resectable, have a significant risk of a positive surgical margin and systemic subclinical spread of disease [114]. Thus, neoadjuvant therapy (e.g., chemotherapy +/− radiotherapy) is currently recommended before surgery [115]. Considering the results of the recent Alliance A021501 trial, which failed to demonstrate any benefit of adding SBRT to systemic therapy in the neoadjuvant treatment of BRPC, FOLFIRINOX has been established as the preferred preoperative treatment option for borderline resectable tumors [116].

However, in a large observational study of primary chemotherapy for newly diagnosed patients with localized disease, the resection rate in the subgroup of BRPC approached 25%, with the highest rate being reached in patients < 75 years who received FOLFIRINOX (51%) [105]. Thus, the same principles regarding the use of ART in the LAPC could also be applied to the BRPC. In this context, the use of dose-escalated radiotherapy in BRPC patients has been reported in limited series, but with promising results. In a single-arm, phase 2 clinical trial conducted at the Massachusetts General Hospital, 48 patients with BRPC received FOLFIRINOX for 8 cycles followed by individualized radiotherapy [117]. Notably, IORT was used in the surgical theater, delivering 10 Gy on the surgical bed, and 15 Gy if the tumor was not resected. Among the 32 (67%) patients who underwent surgical resection, 97% had a R0 resection. In resected patients, the median OS was not reached and the median PFS was 48.6 months. Mellon et al. treated 110 BRPC patients with SBRT delivered in five consecutive daily fractions with a median total dose of 30 Gy to the tumor with a SIB to the tumor–vessel interface (TVI) up to 50 Gy [22]. The R0 resection rate was 96%, and the median OS for resected patients was 34.2 months. A phase I trial investigated a TNT approach with FOLFIRINOX followed by dose-escalated SBRT (up to 36 Gy in three fractions to the tumor with 45 Gy SIB to the posterior margin) in BRPC [118]. The R0 resection rate was 66.6% and the median OS for resected patients was not reached. Similarly, in a recent observational study at Verona Hospital University, 88.9% of BRPC patients received surgical resection (R0 resection rate 60%), following multiagent chemotherapy and SBRT [60]. Notably, in these series, the use of non-standard radiotherapy dosing did not jeopardize subsequent surgery.

In conclusion, data regarding the use of ART in the BRPC are limited. However, early experiences have shown promising results in terms of effectiveness and feasibility. Remarkably, the reported R0 resection rates were extremely encouraging, as were the surgical complications, which were no worsened by using non-standard dosing. Ongoing trials are currently exploring for BRPC patients the role of dose-escalated radiotherapy in the context of a total neoadjuvant multimodal strategy [119,120].

## 7. Conclusions

Optimal treatment of LAPC is ever more frequently multimodal. The effectiveness of standard-dose radiotherapy following chemotherapy, as an alternative to or a bridge to surgery, has been questioned at length. In recent years, ablative radiation therapy (ART) has emerged as a concrete strategy to improve survival in LAPC. However, ART is at its inception, and many open questions remain.

First, several strategies for ART have been reported; however, which may be the most appropriate approach to LAPC has yet to be determined. Technical issues for these patients, including contouring, treatment planning, and delivery are relevant, thus ART should be performed in highly specialized centers. In addition, although ablative dosing could be simplistically adopted as a one-size-fits-all strategy, as LAPC represents a spectrum of heterogeneous disease, a personalized approach to the patient should be pursued. In this context, the availability of reliable biomarkers of biological behavior, as well as genomic/transcriptomic characterization and targeting, could lead to the optimal treatment individualization. Lastly, the optimal integration and sequence with chemotherapy, as well as the role of ART in the neoadjuvant setting, must still be defined.

In conclusion, ART represents a promising treatment option that can be used in a multi-step selective therapeutic strategy, aiming to optimize oncological outcomes in LAPC. ART has been demonstrated to potentially provide local disease control similar to surgery and to improve survival compared with standard-dose radiotherapy. Well-conducted prospective and randomized studies are necessary to draw definitive conclusions.

## Data Availability

Not applicable.
